# Design, Fabrication and Characterization of Multi-Frequency MEMS Transducer for Photoacoustic Imaging

**DOI:** 10.3390/mi17010122

**Published:** 2026-01-17

**Authors:** Alberto Prud’homme, Frederic Nabki

**Affiliations:** Department of Electrical Engineering, École de Technologie Supérieure, Montreal, QC H3C 1K3, Canada

**Keywords:** MEMS transducers, photoacoustic imaging, multi-frequency transducer, piezoelectric devices, ultrasonic transducer, biomedical imaging

## Abstract

This work presents the design, fabrication, and experimental characterization of microelectromechanical system (MEMS) ultrasonic transducers engineered for multi-frequency operation in photoacoustic imaging (PAI). The proposed devices integrate multiple resonant geometries, including circular diaphragms, floated crosses, anchored cross membranes, and cantilever arrays, within compact footprints to overcome the inherently narrow frequency response of conventional MEMS transducers. All devices were fabricated using the PiezoMUMPs commercial microfabrication process, with finite element simulations guiding modal optimization and laser Doppler vibrometry used for experimental validation in air. The circular diaphragm exhibited a narrowband response with a dominant resonance at 1.69 MHz and a quality factor (Q) of 268, confirming the bandwidth limitations of traditional geometries. In contrast, complex designs such as the floated cross and cantilever arrays achieved significantly broader spectral responses, with resonances spanning from 275 kHz to beyond 7.5 MHz. The cantilever array, with systematically varied arm lengths, achieved the highest modal density through asynchronous activation across the spectrum. Results demonstrate that structurally diverse MEMS devices can overcome the bandwidth constraints of traditional piezoelectric transducers. The integration of heterogeneous MEMS geometries offers a viable approach for broadband sensitivity in PAI, enabling improved spatial resolution and depth selectivity without compromising miniaturization or manufacturability.

## 1. Introduction

Microelectromechanical systems (MEMS) have emerged as a transformative technology for enhancing the performance of photoacoustic imaging (PAI) systems. PAI, as a hybrid imaging modality that merges the optical contrast provided by photoacoustic effects with the deep tissue penetration capability of ultrasound, holds significant promise for a broad spectrum of biomedical applications. It enables high-resolution visualization of vascular structures, tumors, and molecular distributions within biological tissues, rendering it invaluable for both clinical diagnostics and preclinical research. The quality and adaptability of the resulting images are closely tied to the characteristics of the ultrasonic transducers employed to detect the photoacoustic signals. Historically, PAI platforms have depended on conventional ultrasound transducers; however, MEMS-based devices have recently emerged as an attractive alternative offering new functionalities and extended capabilities [1,2].

A fundamental requirement for transducers used in PAI is the ability to deliver both high sensitivity and broad frequency coverage, as the spectral content of photoacoustic signals varies widely with the size, composition, and depth of the absorbing structures. This variability arises from the diverse acoustic responses encountered in biological tissues. To maximize image resolution while maintaining penetration depth, it is necessary to employ transducers capable of efficient operation across multiple frequency bands [3].

Conventional PAI transducers are most often fabricated from piezoelectric materials such as lead zirconate titanate (PZT), which convert the mechanical vibrations of ultrasonic waves into electrical signals. Their operating frequency is typically dictated by the thickness of the piezoelectric layer, which sets the device’s primary resonance. Depending on the target tissue or imaging application, these devices are designed for specific frequency ranges: higher frequencies (20–100 MHz) are suited for shallow imaging with fine spatial resolution, while lower frequencies (1–10 MHz) allow deeper penetration at the cost of reduced resolution [4].

Despite their long-standing utility, conventional piezoelectric transducers encounter inherent constraints in multi-frequency imaging scenarios. The optimal transducer for PAI should detect a broad range of ultrasonic frequencies to accommodate signals generated from varying tissue depths, sizes, and compositions, as well as from different laser excitation conditions. High-frequency ultrasound is required to resolve microvascular networks and other fine structures but is prone to rapid attenuation with depth. Conversely, low-frequency ultrasound enables deep imaging of larger anatomical features but lacks the resolution needed for smaller targets. Although modern piezoelectric transducers can achieve fractional bandwidths exceeding 70%, enabling broader frequency coverage, their peak sensitivity typically remains centered around a single operating frequency. Achieving wideband sensitivity necessitates careful optimization of matching layers, backing materials, and the piezoelectric composition [5]. While advanced fabrication and piezocomposite materials have enabled more compact and high-density arrays for real-time volumetric imaging, these improvements are less effective when extreme miniaturization or unconventional geometries are required—conditions where micromachined ultrasound transducers are increasingly advantageous [6,7].

In practice, conventional systems often employ multiple transducers or array elements tuned to distinct frequency ranges to overcome the trade-off between penetration depth and resolution, increasing both system cost and mechanical complexity. Narrowband devices, in particular, face challenges in capturing the full spectral diversity of photoacoustic signals from complex tissue environments containing multiple coexisting frequency components. This limitation reduces versatility and restricts performance in dynamic, multi-layered, or heterogeneous tissue contexts. Since the ultrasonic signals generated by laser-induced optical absorption span a wide range of frequencies, their spectral content depends strongly on absorber size and composition [8]. High frequencies are ideal for resolving minute structures such as capillaries or cellular features, yet they attenuate quickly with propagation through tissue. Low frequencies penetrate more deeply to visualize large-scale features such as organs or tumors but compromise spatial resolution, potentially obscuring fine morphological details [9].

For comprehensive PAI, where both superficial and deep targets must be imaged within a single acquisition, transducers must be capable of detecting across this wide frequency range. Such capability allows simultaneous visualization of superficial vascular details and deeper structural pathologies. Multi-frequency detection further enables advanced modalities such as spectroscopic PAI, in which different tissue components are discriminated based on their unique optical absorption and acoustic signatures at different frequencies. This approach supports tissue composition analysis, molecular marker detection, and differentiation between normal and diseased regions. Without such broadband access, the diagnostic potential of PAI is inherently limited.

MEMS ultrasonic transducers offer a compelling solution by enabling multi-frequency operation through geometrically engineered designs that incorporate multiple mechanical resonances within a miniaturized footprint [10]. Unlike conventional transducers that operate predominantly at a single thickness mode, MEMS devices can be fabricated with suspended membranes, cantilever beams, or composite architectures that support multiple vibrational modes. This allows detection of ultrasonic signals across a broad frequency spectrum without a substantial loss in sensitivity [11]. The inherent broadband nature of such devices facilitates simultaneous acquisition of high-resolution superficial data and deeper structural information, enhancing axial resolution and penetration depth within a single imaging cycle.

The scalability and precision of MEMS microfabrication make it possible to design transducers with tailored higher-order vibrational modes and extended frequency responses. These characteristics are particularly advantageous for spectroscopic PAI, which leverages frequency-dependent acoustic responses to perform functional and molecular imaging beyond purely structural assessment. Additionally, the small form factor of MEMS devices facilitates integration with optical delivery systems and on-chip signal processing electronics, enabling the development of compact, high-performance imaging probes.

This work addresses the design, fabrication, and characterization of MEMS piezoelectric ultrasonic transducers specifically engineered for multi-frequency operation in PAI. The research targets the challenge of achieving broad spectral sensitivity to optimize both resolution and penetration depth for imaging biological samples of varying sizes and depths. By exploiting multi-resonant MEMS architectures, the approach aims to overcome the limitations of conventional piezoelectric designs, enhancing sensitivity, adaptability, and image quality for biomedical imaging applications such as vascular mapping, molecular characterization, and pathological assessment.

## 2. System Integration

Given that the primary objective of this work is the design and characterization of complex MEMS microstructures capable of compensating for the inherently narrow frequency response of individual resonators, the proposed approach focuses on integrating multiple resonant geometries within a single device. This strategy is intended to overcome the limitations of conventional MEMS structures and to enable their potential application in photoacoustic imaging (PAI) systems as viable alternatives to traditional ultrasonic probes. Furthermore, the compact form factor of such devices makes them promising candidates for internal imaging microprobes, where both miniaturization and broad frequency coverage are essential.

To achieve these objectives, a series of MEMS-based microstructures were designed and fabricated, incorporating diverse geometrical configurations and complex mechanical arrangements. The overarching design goal was to maximize the number and spectral distribution of resonant frequencies on a single chip, thereby enabling broadband response characteristics suitable for high-resolution PAI.

**Figure 1:**
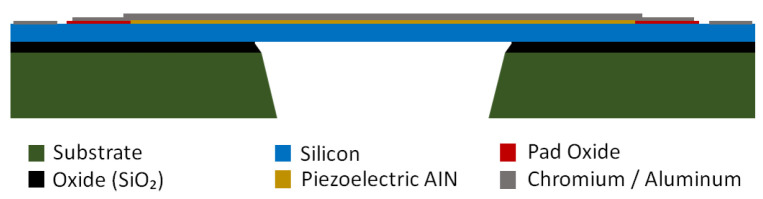
Cross-section of the micro-structures.

The devices were fabricated using the MEMSCAP (Crolles, France) PiezoMUMPs process as shown in the Figure 1, starting from a double-side-polished SOI wafer (10 µm device silicon/1.0 µm buried oxide/400 µm handle). The device silicon is surface-doped to form the bottom electrode. A thermal oxide isolation layer (PADOXIDE, 0.20 µm) is then grown and patterned to provide electrical isolation while opening contact regions where required. Next, the piezoelectric AlN film (PZFILM, 0.5 µm) is deposited and patterned. The Cr/Al top electrode and routing layer (PADMETAL, 20 nm Cr beneath 1.0 µm Al) is subsequently deposited and patterned. The device silicon is then etched by DRIE down to the buried oxide to define the structures. For release, a temporary front-side polyimide protection layer is applied, and the substrate is etched from the backside (TRENCH) by DRIE through the handle wafer, followed by a wet oxide etch to remove the buried oxide in the trench-defined regions and release the suspended structures.

All geometries developed in this work were designed in strict accordance with the dimensional tolerances and design rules of the chosen MEMS fabrication process. These constraints directly influence operational performance, affecting sensitivity, resonance characteristics, and bandwidth.

The following section presents the four evaluated microstructure designs, together with the first-mode resonance frequencies obtained from COMSOL v5.2 Multiphysics simulations, corresponding to the modes with the highest displacement amplitude. Higher-order resonant modes are also illustrated graphically. The experimental frequency responses were measured in air at atmospheric pressure using a Polytec OFV-534 vibrometer operated in closed loop with a Polytec OFV-2570 controller (Waldbronn, Germany). The detailed results for each design are presented in the subsequent section.

### 2.1. Circular Diaphragm-Based MEMS Transducer

**Figure 2:**
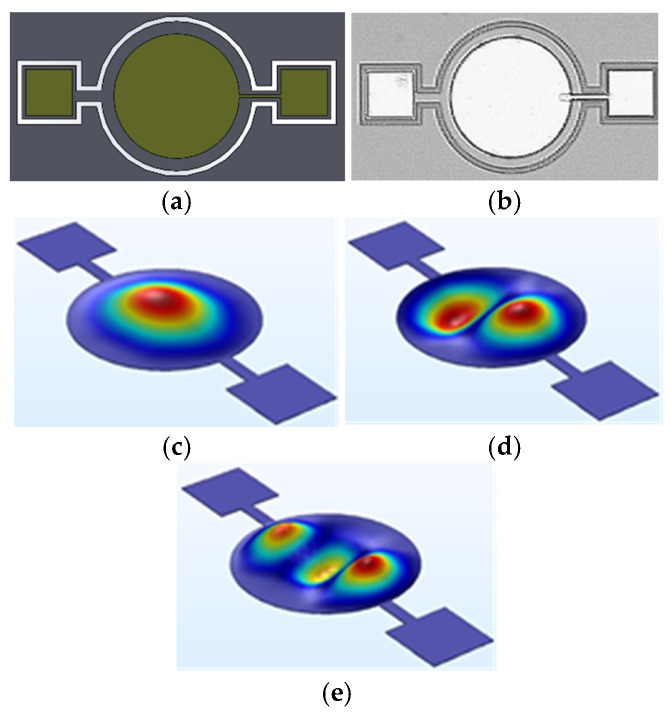
Circular diaphragm micro-structure at (**a**) Device CAD design, (**b**) Microscope image of the fabricated device, (**c**) Mode 1, (**d**) Mode 2 and (**e**) Mode 3.

The first structure fabricated was a conventional circular diaphragm with a diameter of 300 µm, shown in Figure 2. This configuration represents the most basic and widely adopted geometry in MEMS acoustic sensing due to its fabrication simplicity, structural symmetry, and relatively high mechanical efficiency. While the circular diaphragm offers favorable sensitivity, it inherently exhibits a high quality factor (Q), producing a sharply defined resonance peak. Such a narrowband response is suboptimal for photoacoustic imaging (PAI), where a broad frequency range is essential to achieve both high spatial resolution and depth-resolved imaging performance.

Finite element simulations predicted a first-mode resonance frequency of 1.85 MHz, corresponding to the fundamental vibrational mode of the diaphragm. This value was obtained under vacuum boundary conditions, assuming linear elastic behavior of the structural materials. Although the diaphragm supports multiple resonant modes, higher-order responses are typically of very low amplitude for this geometry. Consequently, most of the transduction occurs at the fundamental frequency, and the pronounced Q factor effectively confines the operational bandwidth to a narrow spectral region. This limitation significantly reduces its suitability as a receiver for broadband PAI applications.

To address this constraint, complex geometries were designed with the goal of broadening the frequency response and enabling multiple high-amplitude resonance modes. Such designs are expected to enhance sensitivity across a wider spectral range, improving adaptability to the varying frequency content of photoacoustic signals generated at different tissue depths and with different optical absorption contrasts. This multi-frequency behavior increases the potential utility of the transducer in PAI, allowing simultaneous imaging of fine superficial structures and deeper tissue layers.

### 2.2. Floating Cross-Structured MEMS Transducer

**Figure 3:**
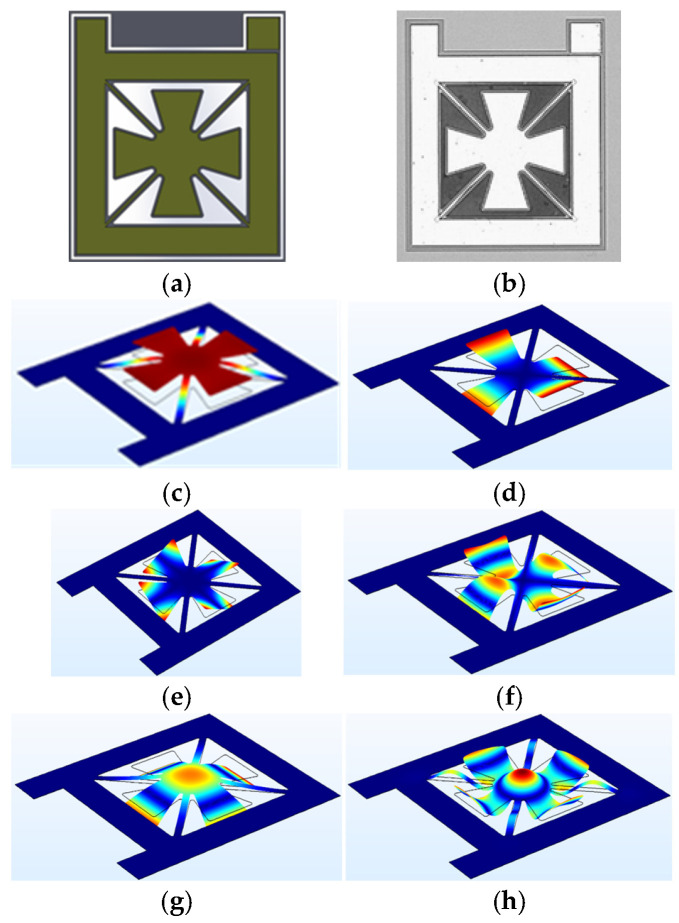
Floated cross micro-structure at (**a**) Device CAD design, (**b**) Microscope image of the fabricated device, (**c**) Mode 1, (**d**) Mode 2, (**e**) Mode 3, (**f**) Mode 4, (**g**) Mode 5 and (**h**) Mode 6.

The next microstructure design focuses on the integration of multiple cantilevers, suspended membranes, and a central region specifically engineered to exhibit high vibrational amplitude. This configuration, referred to as the floated cross, was developed to promote multimodal resonance behavior by incorporating mechanically distinct substructures—each with different stiffness, mass, and boundary conditions—into a single continuous architecture. As shown in Figure 3, the device consists of a central membrane region suspended by four lateral arms, each partially mechanically decoupled from the substrate to enhance dynamic displacement range and allow for independent modal contributions.

The design strategy aims to achieve frequency diversification by exploiting differences in mechanical impedance and geometric scaling between the central and peripheral elements. In this arrangement, the central membrane primarily dictates the low-frequency response, whereas the cantilever-like extensions generate higher-frequency resonances. Finite element simulations predict the first resonance mode, localized within the central membrane, at approximately 300 kHz. The cantilever arms reach their maximum displacement amplitude at a substantially higher frequency of around 800 kHz.

Owing to its geometrical complexity and the interplay between multiple vibrational domains, the floated cross is expected to produce a rich spectrum of higher-order resonant modes. These may involve hybrid coupling between the central membrane and peripheral cantilevers, resulting in spatially distributed and temporally overlapping displacement fields.

Such modal superposition facilitates broadband spectral coverage, enabling the transducer to respond effectively to a wider range of acoustic frequencies. This broadband behavior is particularly beneficial in photoacoustic imaging, where signals generated by heterogeneous tissue structures contain both low- and high-frequency components. By capturing this broad spectrum, the floated cross enhances the ability to resolve superficial microstructures while simultaneously detecting deeper features with high spatial fidelity.

### 2.3. Cross-Structured MEMS Transducer

**Figure 4:**
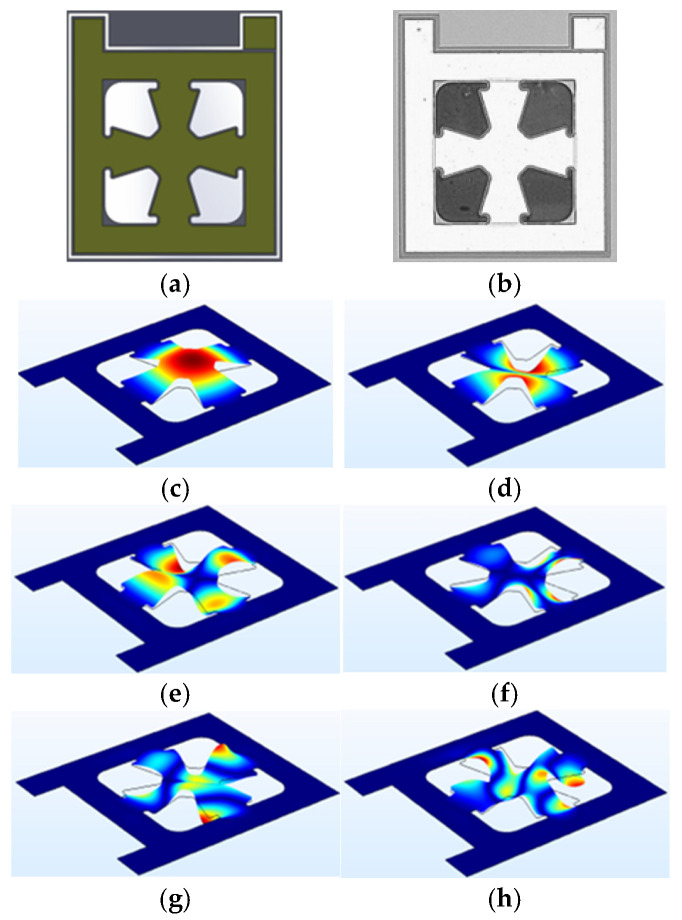
Cross micro-structure at (**a**) Device CAD design, (**b**) Microscope image of the fabricated device, (**c**) Mode 1, (**d**) Mode 2, (**e**) Mode 3, (**f**) Mode 4, (**g**) Mode 5 and (**h**) Mode 6.

The next microstructure design incorporates a central suspended membrane supported by four symmetrically arranged, inward-pointing anchor arms, forming the configuration referred to as the cross microstructure. This geometry, illustrated in Figure 4, is engineered to promote a concentrated vibrational response in the central region, while the surrounding support structure minimizes constraints on the membrane and contributes to higher torsional frequency responses. Unlike previous designs employing elongated and mechanically distinct cantilevers, this structure adopts a simplified yet effective approach to achieving broadband vibrational behavior through a centrally suspended membrane connected by four narrow anchor arms. Finite element simulations indicate that the dominant resonance mode is localized within the central membrane, with a peak response at approximately 275 kHz, corresponding to its fundamental flexural oscillation.

Despite its relatively simple geometry, the cross microstructure demonstrates a notable ability to support multiple higher-order modes across a broad spectral range. This capability is attributed to the dynamic behavior of the anchor arms, which can undergo both bending and torsional deformations under acoustic excitation. Functionally, these arms act as short cantilevers, introducing additional resonances beyond the fundamental mode. Their interaction with the central membrane results in complex mechanical coupling, producing hybrid vibrational modes that are distributed across the entire structure.

In the context of photoacoustic imaging—where detecting a broad frequency range is essential for resolving features at varying depths—this multimodal response is particularly advantageous. The presence of both low- and high-frequency resonances enables the transducer to respond effectively to ultrasonic signals generated by absorbers of different sizes and compositions within biological tissues. Consequently, even in its compact form, the cross microstructure offers a viable path toward wideband operation, fulfilling the performance requirements of modern PAI systems while maintaining fabrication simplicity and structural robustness.

### 2.4. Cantilevers Combination MEMS Transducer

The final design evaluated consists of a closed-frame architecture incorporating an array of cantilevers with varying lengths and a uniform width. The cantilever lengths range from 115 μm to 25 μm, while the width is consistently maintained at 50 μm for all elements. This configuration was intentionally developed to exploit the distinct mechanical responses associated with each cantilever geometry, thereby generating a dense and well-distributed set of resonance modes spanning a broad frequency range.

Each cantilever is capable of supporting multiple vibrational modes—both flexural and torsional—arising from differences in length and associated stiffness. Finite element simulations predict that the first resonance mode of the longest cantilever occurs at approximately 145 kHz, corresponding to a low-frequency flexural mode with high displacement amplitude. In contrast, the shortest cantilever exhibits its primary resonance well above 10 MHz, demonstrating the ability to achieve an exceptionally wide spectral span within a compact footprint.

In addition to these extremes, multiple intermediate resonances were identified and confirmed, with prominent modes observed at approximately 2.1 MHz, 2.6 MHz, and 5.5 MHz. These arise from combinations of flexural and torsional oscillations distributed across the cantilever array, producing complex modal patterns with spatially varying displacement amplitudes. This hybrid vibrational behavior is a key contributor to the broadband operational characteristics of the device.

The multi-modal, multi-frequency response achieved through this architecture is particularly advantageous for photoacoustic imaging applications, where sensitivity to a wide spectrum of ultrasonic frequencies is essential for capturing detailed anatomical and functional information across varying tissue depths and compositions. Due to the high density of active modes, a detailed graphical representation of the device’s frequency response is provided in the Results section.

**Figure 5:**
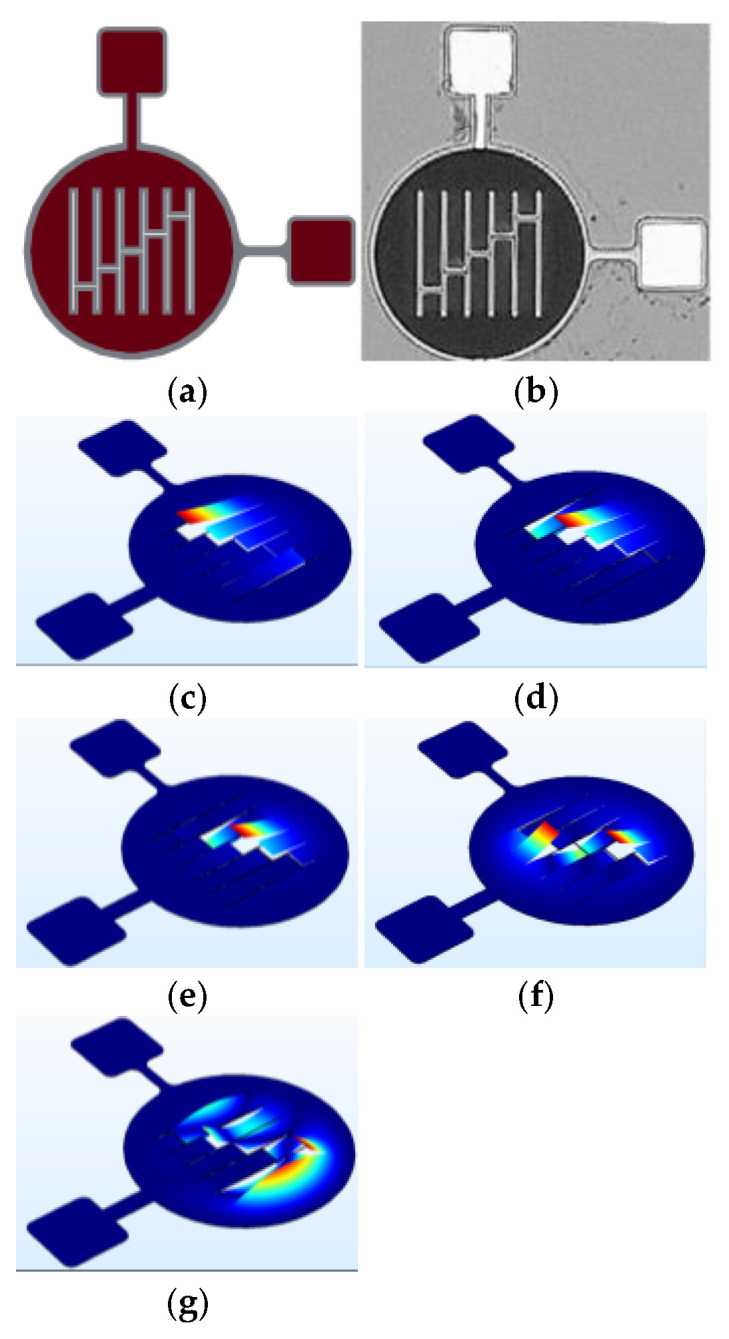
Cantilever-combination microstructure: (**a**) CAD layout; (**b**) optical micrograph of the fabricated device; (**c**–**g**) fundamental out-of-plane mode shapes for arms 1–5, respectively.

As illustrated in Figure 5, the resonance modes corresponding to individual cantilevers are distributed throughout the frequency spectrum. The deliberate variation in cantilever lengths ensures that each element exhibits unique resonance characteristics, resulting in a dense and diversified modal distribution. It is evident that while one cantilever may be vibrating in its fundamental (first-order) flexural mode, another—shorter or stiffer—cantilever may simultaneously resonate at a higher frequency in either a higher-order flexural or torsional mode. This asynchronous activation of different cantilevers produces multiple localized oscillations occurring concurrently within the structure, with each contributing a distinct frequency component to the overall response. The collective effect of these independent yet coexisting modes enables the transducer to operate effectively over an exceptionally broad frequency range.

It can be observed that while one cantilever is vibrating in its fundamental (first-order) flexural mode, another—shorter or stiffer—cantilever may simultaneously resonate at a higher frequency, either in a higher-order flexural mode or in a torsional mode. This asynchronous activation of different cantilevers leads to multiple localized oscillations occurring concurrently within the device, each contributing to a specific frequency component in the overall response. The combination of these independent yet coexisting vibrational modes allows the transducer to operate effectively across a broad frequency range. Available for this study did not permit submerged operation. As a result, all data presented here were collected in air.

## 3. Measurements Results

The vibrational responses of the fabricated MEMS microstructures were experimentally characterized using a Polytec (Waldbronn, Germany) OFV-534 laser Doppler vibrometer (LDV) in combination with an OFV-2570 controller. For membrane-based devices, the LDV spot was positioned at the center of the suspended membrane/diaphragm. For the cantilever-array device, the LDV spot was positioned at the free end (i.e., tip) of the cantilever under test. All measurements were performed at standard ambient temperature (approximately 22 °C) and atmospheric pressure. While practical photoacoustic imaging (PAI) applications typically require immersion in a liquid medium—such as water or a higher-density acoustic coupling fluid—to ensure efficient acoustic transmission, the measurement setup

It is important to note that operating in air influences both resonance frequency and damping. Air loading is significantly lower than fluid loading, which leads to higher measured Q-factors and slight upward frequency shifts compared to immersed conditions. In a water-immersed scenario, viscous and acoustic loading would be expected to reduce Q-factors, broaden resonance peaks, and shift resonances to slightly lower frequencies. These effects are discussed in detail in the Discussion section, where the practical implications for PAI are considered.

**Figure 6:**
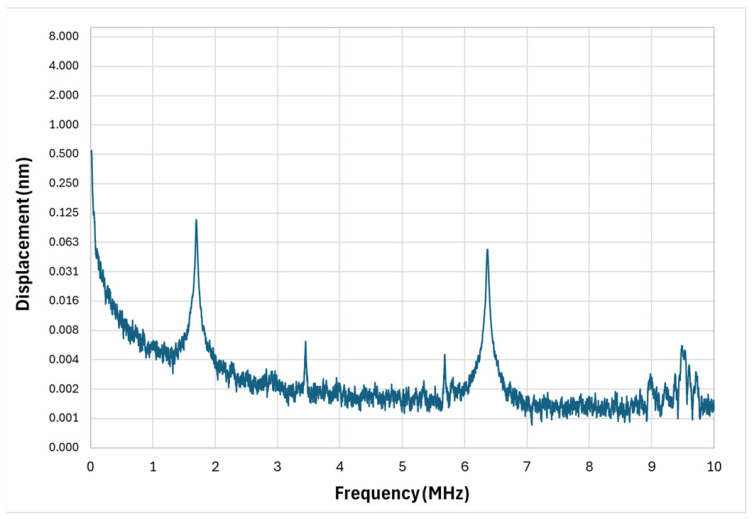
Circular diaphragm micro-structure vibrometer response.

The measured frequency response of the circular diaphragm microstructure is presented in Figure 6. As predicted by simulations, the spectrum is dominated by a single strong resonance corresponding to the fundamental flexural mode.

The experimental fundamental frequency was measured to be 1.69 MHz, closely matching the FEM-predicted value, and the Q-factor was extracted as approximately 268. The Q-factor was obtained from the measured frequency-response curve using the half-power (−3 dB) bandwidth method, as computed automatically by the LDV system software v5.1 during the frequency sweep.

This high Q-factor confirms that the diaphragm experiences very low mechanical damping in air, concentrating vibrational energy into a narrow spectral window. Such behavior is highly beneficial for applications that require frequency stability or selective detection—such as MEMS-based oscillators, resonant sensors, or narrowband ultrasound applications.

However, in PAI the narrow spectral bandwidth is a disadvantage. Broadband photoacoustic signals generated in tissue can extend from a few hundred kilohertz to beyond tens of megahertz, depending on absorber size and composition. A sharply peaked response centered at 1.69 MHz means that much of this information is not captured, resulting in reduced axial resolution and incomplete depth profiling. While higher-order modes are present in the spectrum, their displacement amplitudes are significantly weaker—often below detectable thresholds for practical PAI—making the circular diaphragm effectively a single-frequency device. This confirms the need for alternative geometries that can sustain multiple, high-amplitude resonances over a broader range.

**Figure 7:**
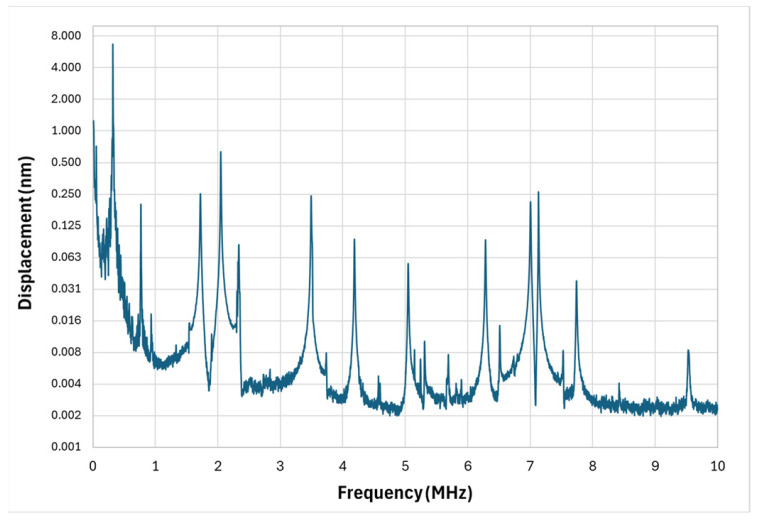
Floated cross micro-structure vibrometer response.

The floated cross design, whose measured frequency response is shown in Figure 7, was developed to introduce spectral diversity by combining a central membrane with partially decoupled cantilever-like arms. Experimentally, this structural complexity is reflected in the large number of observed resonance peaks.

The primary low-frequency resonance was measured at 318 kHz with an exceptionally high Q-factor of 698. While this indicates strong mechanical confinement of energy, the resonance lies below the preferred operational range for most PAI applications. Furthermore, the high Q-factor again narrows the operational window for that specific mode.

More promising, however, are the distributed resonance peaks at higher frequencies, specifically in the 1.5–2.5 MHz and 6.5–7.5 MHz bands. These frequency regions correspond to modes involving both central membrane motion and higher-order bending or torsional vibrations of the arms. In these ranges, displacement amplitudes were significantly higher than in surrounding spectral regions, suggesting that the floated cross can be tuned to emphasize modes relevant for PAI.

Nevertheless, the spectrum is not continuous—there are wide frequency intervals with negligible amplitude, which would result in selective rather than uniform broadband sensitivity. This confirms that while the floated cross geometry achieves multi-modal behavior, optimization is still needed to improve mode density and minimize inactive spectral regions. Adjustments to arm stiffness, membrane thickness, or anchoring geometry could promote overlapping modes and enhance continuous spectral coverage.

**Figure 8:**
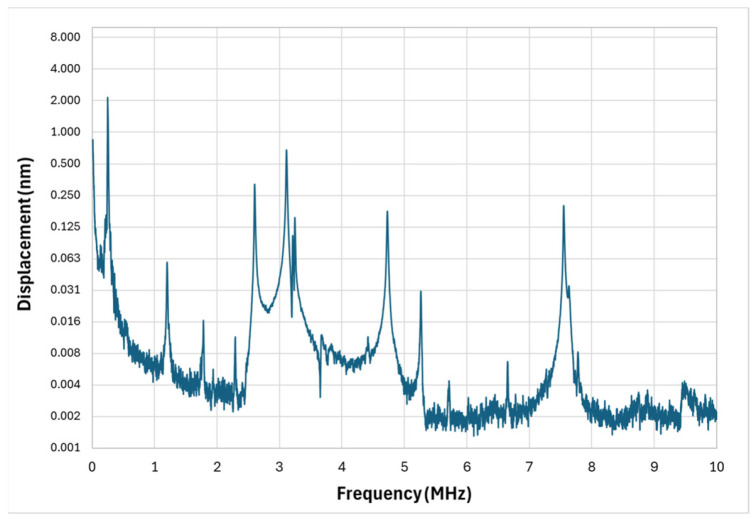
Cross micro-structure vibrometer response.

The cross microstructure’s frequency response is shown in Figure 8. This geometry retains a central suspended membrane but employs four symmetrical anchor arms rather than elongated, partially isolated cantilevers. The design goal was to maintain mechanical simplicity while promoting modal diversity through arm–membrane coupling.

The fundamental resonance of the central membrane was measured at 247 kHz with a Q-factor of 148. Although outside the optimal PAI band, this low-frequency mode is accompanied by multiple higher-frequency responses with significant amplitudes. Most notably, a broad region of activity was observed between 2.5 MHz and 5.2 MHz, suggesting the presence of overlapping modes from both membrane and arm vibrations. A secondary band of strong response was also measured around 7.5 MHz, likely associated with localized high-frequency modes in stiffer or smaller structural regions.

Compared to the floated cross, the cross geometry offers a more continuous distribution of high-frequency modes, despite its simpler form. This may be due to stronger coupling between the arms and membrane, which promotes energy transfer across multiple modes. In PAI terms, this translates to improved ability to capture signals from both superficial and moderately deep tissues without large frequency gaps in sensitivity.

**Figure 9:**
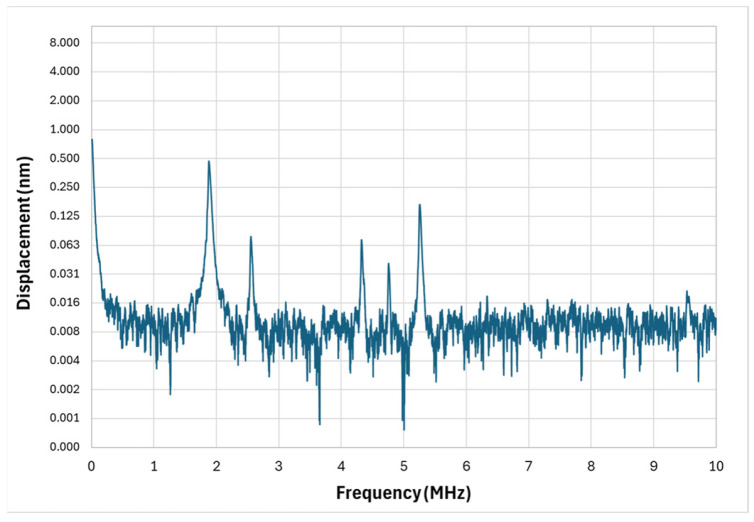
Cantilever combination micro-structure vibrometer response.

The cantilever array’s measured frequency response is presented in Figure 9. This design takes a different approach by using length variation to deliberately spread resonances across a wide spectrum. The longest cantilever exhibited a first-mode resonance at 1.8 MHz with a Q-factor of 204, producing strong displacement amplitude in the lower-megahertz range.

Due to the substantial length variation—from 115 μm to 25 μm—many shorter cantilevers are expected to resonate at frequencies exceeding 10 MHz. However, the LDV measurement bandwidth in this study did not extend into this range, so these high-frequency modes are absent from the presented data. Within the measurable range, multiple secondary peaks were observed at intermediate frequencies, but their high Q-factors resulted in relatively narrow, isolated resonances.

While the array achieves spectral diversity, the lack of modal overlap limits continuous broadband coverage. For optimal PAI performance, overlapping resonances are desirable so that multiple tissue scales and depths can be imaged in the same acquisition. This limitation could be addressed in future designs by slightly altering cantilever lengths to promote modal convergence, introducing controlled damping to broaden peaks, or varying cantilever widths to encourage hybrid mode formation.

## 4. Discussion

The design and experimental characterization of the proposed MEMS transducers clearly demonstrate the potential of multi-resonant microstructures for broadband photoacoustic signal detection. Traditional MEMS diaphragms, while mechanically efficient and straightforward to fabricate, inherently exhibit high Q-factors and narrow frequency responses, as confirmed by the vibrometer measurements of the circular diaphragm. Despite its sharp resonance at 1.69 MHz with a Q-factor of 268, this configuration is fundamentally limited in resolving broadband photoacoustic waveforms, particularly for tissues containing absorbers of varying sizes and depths that emit across a wide spectrum of ultrasonic frequencies.

The alternative geometries developed in this work—the floated cross, the cross configuration, and the multi-cantilever array—exhibited significantly broader frequency coverage enabled by their multimodal vibrational behavior. By integrating multiple distributed resonators within a single structure, these designs generated overlapping resonance modes across the low-megahertz regime, a spectral range highly relevant to photoacoustic imaging in biological tissues. The floated cross demonstrated measurable vibrational activity in the 1.5–2.5 MHz and 6.5–7.5 MHz bands, supporting the hypothesis that hybrid structures composed of membranes and suspended arms can effectively increase modal density. However, the central mode at 318 kHz with a very high Q-factor of 698 illustrates a trade-off between spectral reach and modal uniformity, as significant amplitude gaps remain that reduce detection uniformity across the full acoustic spectrum. The cross configuration provided a more balanced compromise between fabrication simplicity and frequency diversity. Although its fundamental resonance at 247 kHz lies outside the optimal imaging band, its enhanced performance between 2.5 MHz and 5.2 MHz, together with activity around 7.5 MHz, underscores its suitability for detecting signals originating from both superficial and moderately deep absorbers. The mechanically compliant arms contribute higher-order torsional and flexural modes, effectively broadening the response without introducing excessive structural complexity, making this configuration a strong candidate for future MEMS PAI receivers targeting moderate resolution and penetration depth with low fabrication overhead.

The most spectrally dense structure was the cantilever array, which used systematically varied lengths from 115 μm to 25 μm to achieve discrete but widely spaced resonant peaks ranging from sub-megahertz to beyond 10 MHz. Observable activity was centered around 1.8 MHz, with several secondary modes up to 5.5 MHz. The asynchronous activation of individual cantilevers during acoustic stimulation created a spatially distributed and frequency-diverse response profile. However, the modal isolation, evident in the lack of overlap between resonances, resulted in a comb-like spectral response that would benefit from design modifications aimed at lowering Q or promoting mode coupling. Such refinements could include introducing geometrical asymmetries or passive damping elements, such as thin polymer layers or fluidic loading, to generate broader and flatter frequency bands more suitable for broadband photoacoustic detection.

All characterizations were conducted in air at atmospheric pressure, which is not representative of the intended operational environment. Immersion in water or a medium-density liquid will affect both resonant frequencies and quality factors due to acoustic loading and viscous damping. The measured Q-factors in air are therefore expected to overestimate actual values in fluid immersion, potentially resulting in broader frequency responses during real-world operation. Acoustic impedance mismatches between the device surface and biological tissue may also introduce additional damping mechanisms, which could suppress or shift resonance modes, particularly in higher-order responses. These effects, while not captured in the present measurements, are likely to enhance functional bandwidth and improve mode uniformity in practical settings.

While the focus of this study was on mechanical displacement and vibrational amplitude as proxies for transducer sensitivity, future work should address electromechanical coupling factor measurements and photoacoustic reception testing in tissue-mimicking phantoms to fully assess performance under realistic conditions. The spatial distribution of each mode is also an important factor, as it determines the effective acoustic aperture and angular response, both of which strongly influence image reconstruction fidelity and depth resolution in PAI systems.

Due to their small physical footprint, the fabricated MEMS transducers offer the possibility of integrating multiple distinct geometrical designs laterally on the same substrate, creating a heterogeneous transducer array in which each element exhibits a unique and complementary vibrational signature. This spatial multiplexing of functionally diverse microstructures can dramatically enhance spectral coverage, as the combined frequency response of the array encompasses a broader acoustic bandwidth than any individual element alone. By leveraging an ensemble of transducers, each with its own engineered resonance profile, it becomes possible to achieve passive spectral decomposition at the sensor level, improving both spatial resolution and penetration depth without the need for active frequency tuning or complex post-processing. This approach is particularly well aligned with the constraints of endoscopic or catheter-based imaging platforms, where device volume and power budgets are tightly constrained and where large-scale arrays or wideband electronics may be impractical.

**Figure 10:**
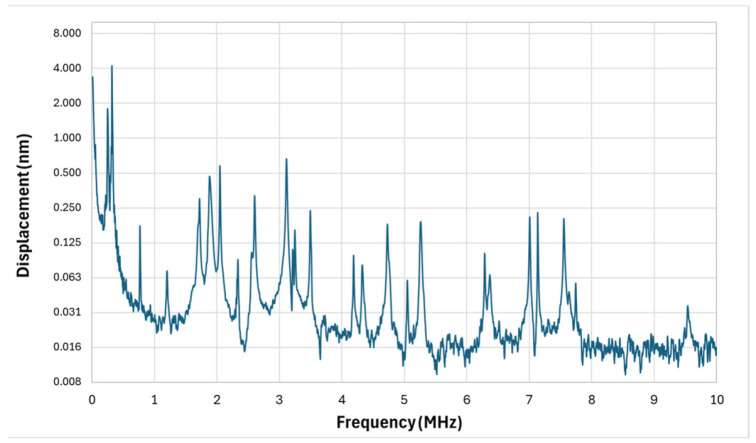
Composite LDV displacement response obtained by normalizing each spectrum to its maximum displacement amplitude over the measured frequency range and superposing the normalized spectra; shaded bands highlight increased resonance overlap at 1.5–3.5 MHz and 6.25–7.75 MHz.

Figure 10 synthesized composite spectral response obtained by combining the measured frequency responses of the circular diaphragm, floated cross, cross microstructure, and cantilever array. Shaded regions (1.5–3.5 MHz and 6.25–7.75 MHz) correspond to frequency bands with enhanced modal density due to overlapping resonance modes from heterogeneous geometries. These regions align with the spectral characteristics of photoacoustic signals from absorbers of varying sizes and depths.

Comparison with prior work, as summarized in Table 1, shows that most reported MEMS ultrasonic transducers, particularly piezoelectric micromachined ultrasonic transducers (pMUTs), rely heavily on circular membrane geometries.

While these designs are advantageous for their modeling simplicity and favorable sensitivity in narrowband applications, their high-Q resonances limit their effectiveness in broadband or time-critical scenarios. The results here underscore the need for alternative geometries or additional damping strategies when designing MEMS transducers for PAI. Potential approaches include non-circular membrane shapes, the incorporation of damping layers, and structural optimization to control anchor loss and air damping. Such strategies could help tailor the Q-factor and bandwidth to match specific application requirements.

Although the measurements in this work were performed in air, the results provide a clear indication of the relative strengths and weaknesses of each geometry and highlight the potential of heterogeneous MEMS designs to deliver the broadband operational characteristics required for advanced photoacoustic imaging.

A practical path to deploy the proposed multi-frequency concept in photoacoustic imaging is through a heterogeneous array architecture, implemented either by spatially interleaving different unit-cell geometries across the aperture or by tiling sub-arrays of distinct geometries. In this approach, each resonator type contributes sensitivity over its dominant frequency region, and the overall detector can be made to operate as a single effective broadband device by combining channels after calibration. Specifically, per-element (or per-type) transfer functions/impulse responses can be measured, followed by gain normalization and phase alignment (where applicable), and band-aware weighting (or frequency-domain stitching) to construct a composite broadband response consistent with the complementary resonance distributions shown in Figure 10.

When multiple resonators of different types are integrated on the same substrate, cross-talk may arise through mechanical coupling via substrate-borne elastic waves and shared anchors/support regions, as well as through electrical coupling via routing, pads, and parasitic feedthrough to the front-end. Mechanical coupling can be mitigated by increasing isolation between neighboring elements (e.g., spacing and isolation trenches/kerfs), minimizing shared support regions, and incorporating localized damping/backing strategies to suppress substrate/anchor modes. Electrical coupling can be reduced through careful interconnect layout, grounded shielding/guard routing, and appropriate low-noise front-end readout design. A quantitative evaluation of these effects, particularly under fluid loading and final packaging, which are expected to shift resonance frequencies and modify damping/Q-factor, is an important next step toward full system-level validation and is outside the scope of the present study.

## 5. Conclusions

This work demonstrates the feasibility of engineering MEMS-based ultrasonic transducers with broadband and multi-frequency response characteristics for photoacoustic imaging applications. Through a comparative analysis of four distinct geometries—circular diaphragm, floated cross, cross-anchored membrane, and multi-cantilever array—it has been shown that structural complexity and modal diversity are critical to extending frequency coverage and improving transducer adaptability to the spectral demands of photoacoustic signals. While conventional circular diaphragms are constrained by a single narrowband resonance, hybrid and distributed microstructures can achieve substantial spectral expansion through tailored mechanical coupling and geometric differentiation.

Future work will focus on refining these designs for immersion operation, optimizing fabrication processes to improve mechanical symmetry and electrical integration, and evaluating photoacoustic sensitivity under in situ optical excitation. The integration of such MEMS transducers into compact imaging arrays offers significant potential for high-resolution, real-time biomedical imaging, particularly in scenarios where spatial selectivity and penetration depth must be balanced without compromising device miniaturization or power efficiency.

## Figures and Tables

**Table 1:** Similar works for MEMS transducers.

Ref.	Shape	Dimension (µM)	Resonator Type	Frequency
[12]	Square	1000 µM	pMUT	2
[13]	Circular	125 µM	pMUT	10
[14]	Variable	Various	pMUT	1–8
[15]	Circular	170 µM	pMUT	0.508
[16]	Circular	100 µM	pMUT	22
[17]	Circular	50 µM	pMUT	8
[18]	Square	250 µM	pMUT	5
[19]	Circular	220 µM	pMUT	1.2
[20]	Circular	160 µM	pMUT	6
This work	Complex	Various	pMUT	1.5–3.5; 6.25–7.75 *

* Composite LDV overlap bands derived from normalized and superposed spectra (Figure 10).

## Data Availability

The original contributions presented in this study are included in the article. Further inquiries can be directed to the corresponding author.

## References

[1] Attia A.B.E., Balasundaram G., Moothanchery M., Dinish U.S., Bi R., Ntziachristos V., Olivo M. (2019). A review of clinical photoacoustic imaging: Current and future trends. Photoacoustics.

[2] Suttikittipong P., Tangkiatphaibun P., Piyawattanametha N., Piyawattanametha W. (2025). Comparison of MEMS-based photoacoustic microscopy in biomedical imaging. Int. J. Optomechatronics.

[3] Ren D., Li C., Shi J., Chen R. (2022). A Review of High-Frequency Ultrasonic Transducers for Photoacoustic Imaging Applications. IEEE Trans. Ultrason. Ferroelectr. Freq. Control.

[4] Manwar R., Islam M.T., Ranjbaran S.M., Avanaki K. (2022). Transfontanelle photoacoustic imaging: Ultrasound transducer selection analysis. Biomed. Opt. Express.

[5] Yang D., Xing D., Yang S., Xiang L. (2007). Fast full-view photoacoustic imaging by combined scanning with a linear transducer array. Opt. Express.

[6] Chan J., Zheng Z., Bell K., Le M., Reza P.H., Yeow J.T.W. (2019). Photoacoustic Imaging with Capacitive Micromachined Ultrasound Transducers: Principles and Developments. Sensors.

[7] Chen B., Chu F., Liu X., Li Y., Rong J., Jiang H. (2013). AlN-based piezoelectric micromachined ultrasonic transducer for photoacoustic imaging. Appl. Phys. Lett..

[8] Xu M., Wang L.V. (2006). Photoacoustic imaging in biomedicine. Rev. Sci. Instrum..

[9] Yao J., Wang L.V. (2014). Sensitivity of photoacoustic microscopy. Photoacoustics.

[10] Liao W., Liu W., Rogers J.E., Usmani F., Tang Y., Wang B., Jiang H., Xie H. Piezeoelectric micromachined ultrasound tranducer array for photoacoustic imaging. Proceedings of the 2013 Transducers & Eurosensors XXVII: The 17th International Conference on Solid-State Sensors, Actuators and Microsystems (TRANSDUCERS & EUROSENSORS XXVII).

[11] Jaber N., Ramini A., Hennawi Q., Younis M.I. (2016). Wideband MEMS resonator using multifrequency excitation. Sens. Actuators A Phys..

[12] Yang Y., Tian H., Yan B., Sun H., Wu C., Shu Y., Wang L.-G., Ren T.-L. (2013). A flexible piezoelectric micromachined ultrasound transducer. RSC Adv..

[13] Dangi A., Agrawal S., Tiwari S., Jadhav S., Cheng C., Trolier-McKinstry S., Pratap R., Kothapalli S.-R. (2018). Evaluation of High Frequency Piezoelectric Micromachined Ultrasound Transducers for Photoacoustic Imaging. 2018 IEEE SENSORS.

[14] Zheng Q., Wang H., Yang H., Jiang H., Chen Z., Lu Y., Feng P.X.-L., Xie H. (2022). Thin ceramic PZT dual- and multi-frequency pMUT arrays for photoacoustic imaging. Microsyst. Nanoeng..

[15] Sun S., Wang J., Ning Y., Zhang M. (2022). Air-coupled piezoelectric micromachined ultrasonic transducers for surface stain detection and imaging. Nanotechnol. Precis. Eng..

[16] Lu Y., Tang H., Fung S., Wang Q., Tsai J.M., Daneman M., Boser B.E., Horsley D.A. (2015). Ultrasonic fingerprint sensor using a piezoelectric micromachined ultrasonic transducer array integrated with complementary metal oxide semiconductor electronics. Appl. Phys. Lett..

[17] Lu Y., Tang H.-Y., Fung S., Boser B., Horsley D. (2015). Short-range and high-resolution ultrasound imaging using an 8 MHz Aluminum Nitride PMUT array. 2015 28th IEEE International Conference on Micro Electro Mechanical Systems (MEMS).

[18] Wang J., Zheng Z., Chan J., Yeow J.T.W. (2020). Capacitive micromachined ultrasound transducers for intravascular ultrasound imaging. Microsyst. Nanoeng..

[19] Wang H., Yang H., Chen Z., Zheng Q., Jiang H., Feng P.X.-L., Xie H. (2021). Development of Dual-Frequency PMUT Arrays Based on Thin Ceramic PZT for Endoscopic Photoacoustic Imaging. J. Microelectromechanical Syst..

[20] Sadeghpour S., Zilonova E., Hooge J.D., Kraft M. (2021). A Novel 6 MHz Phased Array Piezoelectric Micromachined Ultrasound Transducer (pMUT) with 128 Elements for Medical Imaging. 2021 IEEE International Ultrasonics Symposium (IUS).

